# Flexible spatial perspective-taking: conversational partners weigh multiple cues in collaborative tasks

**DOI:** 10.3389/fnhum.2013.00618

**Published:** 2013-09-26

**Authors:** Alexia Galati, Marios N. Avraamides

**Affiliations:** ^1^Department of Psychology, University of CyprusNicosia, Cyprus; ^2^Centre for Applied Neuroscience, University of CyprusNicosia, Cyprus

**Keywords:** perspective-taking, spatial memory, intrinsic structure, audience design, common ground, spatial descriptions

## Abstract

Research on spatial perspective-taking often focuses on the cognitive processes of isolated individuals as they adopt or maintain imagined perspectives. Collaborative studies of spatial perspective-taking typically examine speakers' linguistic choices, while overlooking their underlying processes and representations. We review evidence from two collaborative experiments that examine the contribution of social and representational cues to spatial perspective choices in both language *and* the organization of spatial memory. Across experiments, speakers organized their memory representations according to the convergence of various cues. When layouts were randomly configured and did not afford intrinsic cues, speakers encoded their partner's viewpoint in memory, if available, but did not use it as an organizing direction. On the other hand, when the layout afforded an intrinsic structure, speakers organized their spatial memories according to the person-centered perspective reinforced by the layout's structure. Similarly, in descriptions, speakers considered multiple cues whether available a priori or at the interaction. They used partner-centered expressions more frequently (e.g., “to your right”) when the partner's viewpoint was misaligned by a small offset or coincided with the layout's structure. Conversely, they used egocentric expressions more frequently when their own viewpoint coincided with the intrinsic structure or when the partner was misaligned by a computationally difficult, oblique offset. Based on these findings we advocate for a framework for flexible perspective-taking: people weigh multiple cues (including social ones) to make attributions about the relative difficulty of perspective-taking for each partner, and adapt behavior to minimize their collective effort. This framework is not specialized for spatial reasoning but instead emerges from the same principles and memory-depended processes that govern perspective-taking in non-spatial tasks.

## Spatial perspective-taking in collaborative tasks

When coordinating in joint activities, people routinely have to retrieve spatial information from memory and convey it to others. They do so in a range of tasks, such as describing places they have visited, providing driving directions over the phone, or arranging where to meet. Since in many such socially-embedded tasks people often occupy a different spatial viewpoint from their conversational partner, one important empirical question centers on how readily they take their partner's viewpoint into account.

Much of our understanding of people's ability to adopt or maintain non-egocentric spatial perspectives stems from studies using non-interactive tasks (e.g., Carlson-Radvansky and Irwin, 1993; Carlson-Radvansky and Logan, 1997; Mou et al., 2004b). Studies using interactive tasks, on the other hand, identify factors that influence the perspective from which people tend to produce or interpret spatial descriptions (e.g., Schober, 1993; Mainwaring et al., 2003; Tenbrink et al., 2011). However, such studies typically don't examine directly the underlying off-line spatial representations or on-line processes that support perspective-taking (but see Shelton and McNamara, 2004; Duran et al., 2011; Galati et al., 2013).

Thus far, the findings emerging from both interactive and non-interactive tasks suggest that when people select a spatial perspective—whether to describe spatial information or to organize spatial information in memory—they consider various sources of information, including cognitive, contextual, and social factors. For instance, such factors have been shown *individually* to constrain how people organize and maintain spatial information in memory. When learning and remembering a spatial layout, people appear to interpret it in terms of a reference system that maintains spatial relations around a preferred direction (e.g., McNamara, 2003; Mou et al., 2004a), a process analogous to determining its “top.” This preferred direction is influenced by egocentric information, such as one's initially experienced viewpoint (Shelton and McNamara, 2001), representational or environmental information, such as the environment's geometry (Shelton and McNamara, 2001), the symmetry or intrinsic structure of the spatial configuration (Mou and McNamara, 2002; Li et al., 2011), functional features of landmarks in the configuration (Taylor and Tversky, 1992), and even social information available from the visual context, such as their conversational partner's viewpoint (Shelton and McNamara, 2004; Galati et al., 2013).

In this article, we present a framework for how people spontaneously recruit both social and representational information in spatial reasoning. Our view is that people consider all available cues and, upon gauging the relative cognitive demands of perspective-taking on each partner, weigh these cues accordingly to select the perspective that would minimize the pair's joint effort. This view differs from earlier proposals that have acknowledged the contribution of multiple sources of information in spatial reasoning, but have given precedence to representational cues (namely, egocentric experience, Shelton and McNamara, 2001, and the intrinsic structure of the layout, Mou and McNamara, 2002). Our framework emerges from our own work examining the perspectives people adopt in joint tasks both in their spatial descriptions and in their underlying representations supporting those descriptions. This framework allows for predictions for both linguistic and memory performance and can account for how people adapt their encoding, description and coordination strategies as incoming cues become available in range of spatial tasks.

Since our focus here is on collaborative tasks, we begin by reviewing in the next section studies demonstrating the contribution of social cues in spatial perspective-taking. Then, we present evidence from own experimental work demonstrating that people integrate social cues (the availability of partner's viewpoint) with representational ones (their misalignment from their partner's viewpoint or from the configuration's intrinsic structure) to determine the perspective from which to organize information in memory and subsequently describe this information to a partner. Since an assumption of our framework is that partners jointly aim for efficient communication, in a subsequent section, we address whether people's perspective choices are in fact effective, as reflected by the pairs' efficiency and accuracy in the joint task. In the final section, we flesh out in more detail the characteristics of our proposed framework, addressing along the way some of the predictions it affords for a range of spatial perspective-taking tasks. We conclude that people weigh multiple cues to determine the task's cognitive demands on themselves and their partners, and as a result select strategies that are generally effective in facilitating coordination.

## Social cues influence spatial perspective-taking

A growing body of evidence suggests that, when adopting a spatial perspective, people consider different sources of social information, including attributional and contextual cues about their conversational partner. Attributional cues about the partner include pre-existing beliefs or attributions made about the partner based on prior experience, expectations or a stereotype (e.g., believing that the partner is unfamiliar vs. familiar with the environment, believing that the partner is a child vs. an adult). Such cues, if not available in advance, can also be accumulated during the course of the interaction and may even be used to update initial beliefs about the partner (e.g., Brennan et al., 2010). Whereas attributional cues pertain to the partner's cognitive or other intrinsic abilities, contextual cues are not intrinsic to the partner, but are instead visually available in the physical environment and concern the partner's visibility, relative position in space, misalignment, or other relevant external features.

In line with other researchers (Clark and Wilkes-Gibbs, 1986; Schober, 1995; Duran et al., 2011), we propose that, on the basis of social cues, people make inferences about their conversational partner's ability to contribute to the joint task and adapt their perspective-taking behavior accordingly. This view follows from the proposal that, when collaborating, people share responsibility for ensuring mutual understanding and try to minimize their collective effort. This shared responsibility requires one partner to invest greater cognitive effort when appraising that the other partner is likely to find the interaction difficult; such behavioral adjustments are said to follow *the principle of least collaborative effort* (Clark and Wilkes-Gibbs, 1986; Clark, 1996). The evidence we report in the next two subsections, on how individual social cues influence perspective-taking, are broadly compatible with this view.

### Attributional cues about the partner influence spatial perspective-taking

As we have mentioned, one source of social information that shapes perspective-taking arises from the attributions people make about the partner's ability to contribute to the joint spatial task. Such attributions can depend on the status of the partner—for instance, whether the partner is believed to be real, imaginary, or simulated. There is evidence that with imaginary partners, or with partners with whom they cannot interact contingently, speakers are more likely to invest in adopting the partner's perspective. That is, speakers are more likely to use descriptions from the partner's perspective (e.g., “to your right” or “in front of you”) and less likely to use egocentric ones when describing spatial layouts to imaginary partners than to real ones (Schober, 1993). Speakers are also less likely to disambiguate the spatial descriptions they produce when they suspect that their partner is a confederate and does not have real informational needs (vs. a naïve participant) (Roche et al., 2010). This adaptation in perspective holds not only for the production of spatial expressions but for their interpretation as well. When listeners believe that their partner is real (vs. simulated) they are more likely to interpret ambiguous spatial descriptions egocentrically than from their partner's perspective (Duran et al., 2011). Comparable adaptation is found in non-linguistic communication strategies as well: in a “tacit communication game” in which participants could convey their intentions only through graphical means, they spent more time signaling the location of critical information to their partner when they believed they were interacting with a child than with an adult (Newman-Norlund et al., 2009).

Thus, when people believe that their partner cannot coordinate with them contingently or is otherwise less able to, they are more likely to adopt the partner's perspective and invest the effort to convey spatial information to them. And conversely, when people believe that their partner is real and able to coordinate with them contingently, they are more likely to shift the burden of mutual understanding to the partner, producing or interpreting spatial descriptions egocentrically.

Similarly, speakers adapt their spatial descriptions according to their beliefs about the partner's familiarity with the environment pertinent to the task. When speakers describe landmarks to a partner who is likely to be is unfamiliar with them (e.g., Washington Square Park to a non-New Yorker), speakers use more detailed descriptions and are less likely to refer to the landmarks by their proper names than when they interact with partners who are natives of the city (Isaacs and Clark, 1987). Speakers also adapt how they plan and describe routes within environments. When describing routes to a partner who is presumed to be unfamiliar with the environment (vs. for themselves), speakers elaborate their descriptions by using more words and details, refer to more landmarks for orienting, and simplify the routes by navigating along fewer, larger and more prominent streets (Hölscher et al., 2011).

As this last study suggests, the framing of the task as collaborative or intended for an audience, as opposed to a monologic activity, can shape spatial descriptions. In a related study, Mainwaring et al. (2003) demonstrated that speakers were more likely to adopt their own perspective when describing spatial information for themselves, thus bearing the cognitive burden exclusively, whereas they were more likely to adopt their partner's perspective when describing spatial information to a misaligned imaginary partner who presumably bore more of the cognitive burden (see also Schober, 1993).

Moreover, people adapt their perspective-taking behavior not only on the basis of their beliefs about the partner's ability to contribute to the spatial task, but also on the basis of so-called “second order beliefs” about the partner: the speaker's beliefs about what the partner believes about the speaker's viewpoint or abilities. For example, when people believe that their partner doesn't know their spatial viewpoint (and therefore cannot consider their perspective), they are more likely to interpret spatial descriptions from the partner's perspective (Duran et al., 2011).

In addition to social cues that are available a priori (e.g., by being told in advance that the partner is unfamiliar with the environment, or that the partner is a child), people can often discover such cues by accruing relevant evidence as the interaction unfolds. For example, based on their partner's performance and feedback, people can make attributions about their relative spatial skills and thus their ability to advance the joint goals of the task. Schober (2009) demonstrated that perspective adaptation can occur on the basis of local, incremental cues, using preselected pairs of participants that had matched or mismatched spatial abilities. As expected, high-ability speakers were overall more likely to use partner-centered descriptions whereas low-ability speakers were more likely to use egocentric ones. But critically, during the course of the interaction, as high-ability speakers in mixed pairs formed attributions about their low-ability partners they increased their use of partner-centered descriptions, and conversely, low-ability speakers describing to high-ability partners decreased their use of partner-centered descriptions. Similarly, incremental visual cues about progress on the task, such as errors indicating the partner's misunderstanding, can contribute to updating attributions about the partner and lead to appropriate adaptation, such as disambiguating spatial descriptions (Roche et al., 2010). Thus, along with a priori information, incoming information about the partner's ability to contribute to the spatial task can influence perspective-taking.

### Contextual cues relating to the partner influence spatial perspective-taking

In assessing the relative cognitive demands of perspective-taking, people consider not only attributional cues pertaining to the partner's knowledge or ability, but also contextual cues concerning the partner's spatial relation to themselves and other features of the environment. Information that is visually available in the shared environment, what is termed as the partners' physical co-presence, is one of the principal heuristics that people assess in order to establish what they have in common ground and to tailor their behavior accordingly (Clark and Marshall, 1981; Clark and Brennan, 1991).

The visibility between partners is, most obviously, one factor that shapes what is physically co-present and thus influences how people interact in the context of spatial tasks. For example, in a task where partners reconstructed arrangements of lego blocks, pairs who could see each other coordinated differently than those who couldn't: speakers adapted their descriptions contingently as their addressees exhibited, poised, pointed at and oriented blocks, which resulted in more accurate and efficient performance on the task (Clark and Krych, 2004). Similarly, in a task in which pairs were trying to align icons on identical maps displayed on networked computer screens, when the person giving directions lacked visual evidence about their partner's icon movements, they left the initiative to move to the next trial to their partner and went through a lengthier process of verbally checking that they have achieved mutual understanding (Brennan, 2005).

The misalignment between partners is another salient contextual cue that shapes people's attributions about the partner's ability to contribute to the task, making it perhaps the factor most often manipulated in interactive studies of spatial perspective-taking. There's evidence that when pairs jointly reconstruct layouts that are simple or randomly configured, the degree of misalignment between partners influences the perspective of speakers' descriptions. For instance, speakers are more likely to use partner-centered descriptions than egocentric ones when describing layouts to partners who are misaligned rather than aligned with them (Schober, 1993, 1995). A caveat here is that, in these and other experiments (e.g., Mainwaring et al., 2003; Duran et al., 2011), partners were misaligned exclusively by orthogonal offsets (i.e., were at 90°, 180°, or 270°). Because orthogonal perspectives are aligned with one's canonical axes, they may be privileged and thus relatively easily adopted or maintained (see McNamara, 2003; Avraamides et al., 2013). The facilitation of the canonical axes may, thus, account for the similarities in participants' description preferences across different offsets. In our own studies (Galati et al., 2013; Galati and Avraamides, in revision), which will be subsequently described in detail, we have addressed this possibility by including oblique offsets between partners in order to determine when, in fact, perspective-taking is most computational demanding for speakers.

The misalignment between partners also influences the shape of their shared space, which can consequently influence spatial descriptions, even when these descriptions are embedded in a narrative. For instance, speakers adapt the directionality of their gestures that accompany spatial prepositions, such as *in* and *out*, as a function of the shape of the space they share with their conversational partners (Özyürek, 2002). When partners are seated face-to-face *in* and *out* are mapped onto a sagittal axis with respect to the speaker's body, whereas when the partner is seated to the side *in* and *out* are mapped onto a lateral axis. These findings are taken to suggest that, in interaction, spatial concepts are encoded with respect to the partners' shared space (i.e., with *in* corresponding to the “inside” of the shared space).

In sum, people consider various aspects of their relation to their partner within the physical environment, including their partner's visibility, their degree of misalignment, and the shape of their shared space. Upon considering these contextual cues, they adapt their descriptions or coordination strategies accordingly.

### Beyond the influence of isolated social cues

So far we have considered evidence that people consider social cues, either available from the onset of the interaction (e.g., through advance instructions or through the physical environment) or accrued during the course of the interaction (e.g., through the partner's feedback) to make attributions about their partner's ability to contribute to the task.

Overall, when people perceive their partner to be limited in some way, they invest the effort to adopt their partner's perspective or to convey information in a more accessible way. This is the case when they believe that the partner is imaginary (Schober, 1993), a child (Newman-Norlund et al., 2009), or unfamiliar with the environment (Hölscher et al., 2011), when they believe the partner does not know (Duran et al., 2011) or does not share their viewpoint (Schober, 1993, 1995; Mainwaring et al., 2003), when they discover that the partner has worse spatial abilities than they do (Schober, 2009), or cannot provide feedback during the interaction (Shelton and McNamara, 2004; Duran et al., 2011). On the other hand, when people perceive their partner to be less limited, as for example when they interact with a real (or assumed to be real) partner or a partner who can contribute contingently to the interaction, they may not invest as much effort in adopting the partner's perspective and instead rely on that partner to request clarifications, as needed. Together, these studies serve as a compelling demonstration of the principle of least collaborative effort (Clark and Wilkes-Gibbs, 1986; Clark, 1996) in spatial tasks.

However, few studies have addressed directly how social cues are considered alongside other sources of information pertinent to spatial tasks, such as information about the intrinsic structure of the configuration. Since real-world environments are often systematically organized, having axes of symmetry or salient landmarks, when selecting the perspective from which to describe them, speakers likely consider not only their partner's viewpoint but also other representational cues intrinsic to the configurations. Indeed, some of the reviewed studies allude to the possibility that people integrate multiple sources of information, even if this is not examined directly. For example, in Hölscher et al.'s study (2011), a social cue (the partner's assumed familiarity with the environment) influenced the extent to which representational cues (landmarks and other salient features of the environment) were incorporated in route descriptions.

Our research agenda has focused on elucidating precisely how social cues about the conversational partner interact with other sources of information during spatial reasoning. This approach extends the principle of least collaborative effort (Clark and Wilkes-Gibbs, 1986; Clark, 1996), insofar as it places an emphasis on the probabilistic weighing and interaction of social and other cues when assessing collaborative effort. Moreover, our approach focuses not only on clarifying how people combine various sources of information to adapt how to coordinate in spatial tasks, but also how this behavior is supported by the cognitive infrastructure—namely, by spatial memory representations. Relating perspective-taking choices in descriptions to their underlying spatial representations would further bolster the view that partner-specific adaptation in dialog is supported by ordinary cognitive processes acting on memory representations (e.g., Horton and Gerrig, 2002, 2005; Metzing and Brennan, 2003; Pickering and Garrod, 2004). Thus, in our work, we examine how social and representational cues interact to influence, not only speakers' spatial descriptions, but also the preferred perspective around which they organize spatial information in memory (see the next section).

Others have shared our view that, at least with respect to organizing and maintaining spatial information in memory, a number of cues are taken into account. For instance, McNamara and his colleagues have proposed that learning and remembering a spatial layout involves interpreting it in terms of a reference system, whose selection depends on spatial and non-spatial properties of the objects, the structure of the surrounding environment, the observer's egocentric viewpoint, and even verbal instructions (Shelton and McNamara, 2001; Mou and McNamara, 2002). But contrary to these proposals, which ascribe precedence to certain cues as being dominant, such as egocentric experience (Shelton and McNamara, 2001) or the intrinsic structure of the layout (Mou and McNamara, 2002), we consider all available cues to be probabilistically combined upon being weighted according to task-specific demands.

In the context of collaboration, task-specific demands arise from aiming for effective coordination. On the basis of such demands, our framework affords predictions for how different cues are weighted and ultimately whose perspective is selected, whether for organizing spatial information in memory or for descriptions. Our framework also affords predictions about how people make use of cues that become available at different time points of a spatial task, as for example when discovering the partner's viewpoint relative to a configuration only *after* the configuration has been learned. Specifically, our framework assumes a great deal of flexibility in incorporating incoming cues to select a spatial perspective (see also Li et al., 2011). For instance, it predicts that when having to describe from memory a learned configuration, people won't simply select the perspective according to which their spatial memory is organized, but will also take into account new perceptually available cues from the interactive situation.

In the next two sections, we present some of our experimental work, which demonstrates that partners consider multiple cues to assess each other's cognitive demands when encoding and communicating spatial information. In the final section of this article, we describe in more detail our framework for flexible perspective-taking, which qualitatively accounts for our experimental results and affords predictions for other perspective-taking tasks.

## Weighing social and representational cues in spatial perspective-taking

In our work, we have focused on one contextual social cue—the a priori visual availability of the partner's misaligned viewpoint. Our goal was to examine the conditions under which this a priori social cue influences how speakers spontaneously organize spatial information in memory and how they describe it to their partner. In the first study (Galati et al., 2013), we examined whether knowing the partner's viewpoint in advance is, on its own, a sufficient cue to influence speakers' memory and descriptions. In the second study (Galati and Avraamides, in revision), we examined how the availability of the partner's viewpoint may be used in conjunction with another representational cue, the intrinsic structure of the spatial layout, to shape memories and descriptions.

In both studies, in order to clarify how memory representations support perspective-taking behavior, we dissociated the learning of spatial layouts from their description: speakers first learned a spatial layout, had their memory of the layout assessed, and then described it from memory to a partner. Most earlier studies don't address the relationship between memory representations and linguistic choices, as they involve situations in which speakers can see the spatial information they describe (e.g., Schober, 1993, 1995, 2009; Mainwaring et al., 2003), learn the spatial information while simultaneously describing it (Shelton and McNamara, 2004), are instructed to describe spatial information from a particular perspective before their memories are assessed (Shelton and McNamara, 2004), or describe familiar environments whose underlying memory representation is not directly assessed (Hölscher et al., 2011). Dissociating the encoding of spatial information of from its description enables us to determine not only whether advance knowledge of the partner's viewpoint influences speakers' memories and descriptions, but also the extent to which speakers rely on their memories when describing spatial information.

### The influence of the availability of the partner's misaligned viewpoint

**Figure 1:**
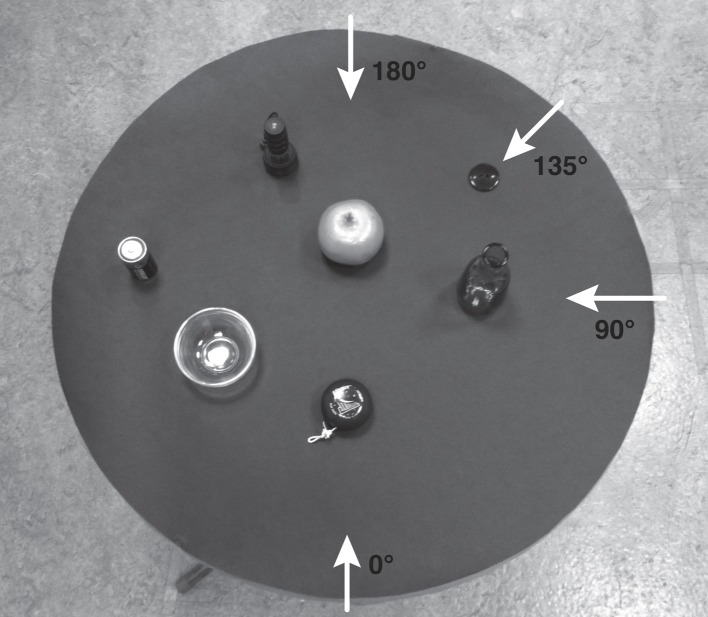
**One of the three seven-object layouts used in Galati et al. (2013),whose configuration was designed to appear seemingly random**. It comprised a battery, a flashlight, a bowl, an orange, a yoyo, a button, and a vase. The arrows represent the Director's viewpoint (0°), and the Matcher's viewpoint when offset by 90°, 135°, and 180°.

**Figure 2:**
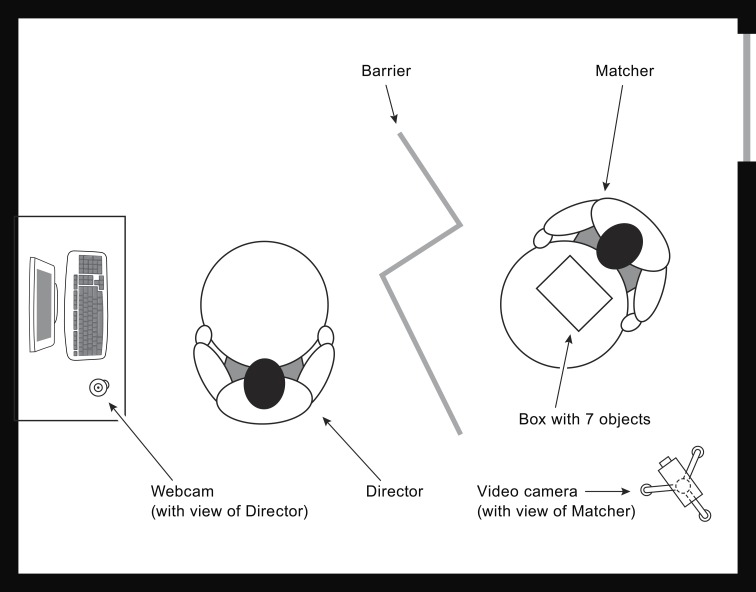
**Set-up of our studies, showing the Director's and Matcher's working stations, and the locations of recording devices**. This example of a description phase illustrates the conditions of Galati et al. (2013) in which Directors and Matchers were misaligned by 135°, and the conditions of Galati and Avraamides (in revision) in which Directors were aligned with the intrinsic structure (with Matchers misaligned by 135°).

In Galati et al. (2013), we asked whether knowing the partner's viewpoint in advance influences speakers' memory and descriptions. In 18 pairs, one participant (the Director) first studied a randomly configured tabletop layout of seven objects (see Figure 1). They later described it from memory to another participant (the Matcher), seated at a separate round table, who reconstructed the layout by following the Director's descriptions (see Figure 2). This took place across three blocks that varied in terms of what Directors knew about their Matcher's viewpoint when studying the layout. In the first block, Directors didn't know that they would later describe the layout to a Matcher, whereas in the subsequent blocks, whose order was counterbalanced across pairs, they either knew they would describe the layout to a Matcher but didn't know the Matcher's viewpoint, or knew the Matcher's viewpoint because the Matcher was co-present in the room during learning, seated at the position they would occupy at the description phase. The degree of misalignment between partners during the description phase, was 90°, 135°, or 180°, and was counterbalanced across the three blocks.

After studying the spatial layout, the Director's memory of it was assessed through two tasks. The first involved *judgments of relative direction* (JRDs), which required imagining a specific location and orientation, and pointing with a joystick to another object from that imagined perspective (e.g., *Imagine being at the vase, facing the orange. Point to the button*.) These JRD trials included eight imagined headings (0°, 45°, 90°, 135°, 180°, 225°, 270°, 315°), whose order was randomized. Performance was assessed in terms of Directors' orientation latency (the time taken to adopt the imagined perspective of the first instruction) and their response latency (the time taken to point to the target identified in the second instruction). Performance on JRDs permits determining the preferred direction participants use to organize the spatial relations in memory (e.g., Kelly et al., 2007). The rationale is that spatial relations specified with respect to the preferred direction can be retrieved from memory more readily than those relations that are not explicitly specified and therefore have to be inferred. Thus, judgments from headings aligned with that preferred direction should show facilitation in terms of the orientation and response latencies.

We found that, when the Matcher's viewpoint was unavailable at study (whether on the first or a subsequent block), Directors encoded spatial layouts egocentrically: they were faster to imagine orienting to and to respond from perspectives aligned with their own. On the other hand, when the Matcher's viewpoint was known in advance, it *was* encoded in memory, showing distinctive processing, at least when Matchers were known to be misaligned by 90° or 135°. When knowing that the Matcher would be at these offsets, Directors took longer to imagine orienting to headings aligned with these known viewpoints. This slower orienting may seem counterintuitive in light of previous findings that speakers can show facilitation for the partner's viewpoint (Shelton and McNamara, 2004). However, in our study, Directors knew that during the description phase they could interact freely with their Matchers and that their respective viewpoints would be mutually known (cf. Shelton and McNamara, 2004), such that the Matcher could bear some of the cognitive burden of perspective-taking. We therefore proposed that our Directors may have not invested the cognitive effort at study to organize spatial relations from their Matcher's viewpoint, but instead encoded their Matcher's viewpoint to use it later, as needed. The longer orientation latencies may therefore reflect a reconstructive process, whereby Directors recalled an episodic representation of their experience at study, which included the location of the Matcher in space, and linked the Matcher's viewpoint to their representation of the layout.

The second memory task provided corroborating evidence that Directors represented the partner's viewpoint in memory. In this task, the Directors drew the spatial layout by indicating the position of each object on a grid circle representing their table. These array drawings allowed us to assess the Directors' memory for the relative positioning of objects and for systematic biases (e.g., Friedman and Kohler, 2003). We found that when Directors knew their Matcher's viewpoint in advance, their drawings showed a reliable rotational bias by approximately 5° toward the Matcher's viewpoint.

Following these memory tasks, Directors described the layout from memory to their Matcher. We examined the distribution of Directors' egocentric (e.g., “*in front of me* is the bracelet”) and partner-centered (e.g., “the battery is *to your right*”) expressions. The distribution of these types of expressions allows for inferences concerning whether an egocentric or partner-centered perspective was predominately in use, and thus reflect Directors' overall description strategies. We found that Directors did adapt their spatial expressions according to what they had known about their Matchers at study (see Table 1). However, knowing the Matcher's viewpoint in advance did not determine on its own the perspective of Directors' descriptions. For instance, when Directors knew their Matcher's viewpoint at study, they didn't simply use more partner-centered expressions during the description. Instead, they made strategic choices upon considering the demands of perspective-taking on themselves and their Matchers.

**Table 1:** **Means (and standard deviations) of the proportions of Director-centered and Matcher-centered expressions produced by Directors describing layouts that were randomly configured (Galati et al., 2013) or with an intrinsic structure (Galati and Avraamides, in revision)**.

	**Director-centered**	**Matcher-centered**
**CONFIGURATION IS RANDOM**		
** Matcher's viewpoint unavailable^a^**		
Misaligned by 90°	0.16 (0.22)	0.28 (0.17)
Misaligned by 135°	0.16 (0.14)	0.23 (0.21)
Misaligned by 180°	0.22 (0.23)	0.18 (0.14)
** Matcher's viewpoint available**		
Misaligned by 90°	0.15 (0.20)	0.26 (0.17)
Misaligned by 135°	0.42 (0.23)	0.08 (0.13)
Misaligned by 180°	0.16 (0.11)	0.24 (0.16)
**CONFIGURATION HAS INTRINSIC STRUCTURE**		
** Matcher's viewpoint unavailable**		
Aligned with Director	0.34 (0.20)	0.09 (0.16)
Aligned with Matcher	0.00 (0.00)	0.45 (0.05)
Aligned with Neither	0.27 (0.25)	0.10 (0.13)
** Matcher's viewpoint available**		
Aligned with Director	0.17 (0.09)	0.18 (0.16)
Aligned with Matcher	0.05 (0.07)	0.31 (0.31)
Aligned with Neither	0.00 (0.00)	0.29 (0.20)

*For both studies, the distribution of these expressions is shown across the availability of the Matcher's viewpoint at learning (unavailable vs. available) and across the relative positioning of the conversational partners (in Galati et al., 2013, with respect to their misalignment, whereas in Galati and Avraamides, in revision, with respect to their alignment with the intrinsic structure.)*.

a *This combines the two conditions from Galati et al. (2013), in which the Matcher's viewpoint was unavailable: the first block in which Directors didn't know there would be a description phase and a subsequent block in which they did know about the description phase but did not know the Matcher's viewpoint*.

When perspective-taking was relatively easy (at the small offset of 90°), Directors used Matcher-centered expressions more frequently than egocentric ones. When pairs were counteraligned and thus shared a canonical axis, Directors mixed perspectives more frequently, suggesting that they could alternate flexibly between their own and their partner's perspective. When perspective-taking was known to be more computationally demanding for Directors, at the oblique offset of 135°, they were more likely to describe layouts egocentrically, as shown in Table 1. That is, since Directors presumably bore more of the cognitive burden in this task, having to recall spatial relations and convey them to their partner, they opted for their own perspective when perspective-taking was especially demanding for them, letting their partners unpack the spatial mappings of their egocentric descriptions. Explicit agreements between partners to do so did, indeed, happen most often when partners had known in advance they would be offset by 135° relative to the other offsets. Thus, the availability of the partner's viewpoint enabled both interlocutors to mutually recognize when the cognitive demands would be taxing for the person carrying the greatest cognitive load and to adapt their communication strategies in ways that facilitated their coordination (for evidence for this facilitation see the next section, on the Coordination in Spatial Perspective-Taking).

Thus, speakers do not spontaneously use their partner's viewpoint as an organizing direction for their memories when it is available; in our study, Directors didn't show facilitation for their partner's viewpoint (cf. Shelton and McNamara, 2004). But despite not using the partner's viewpoint as an organizing direction, speakers do represent that viewpoint in memory; this was evidenced by the Directors' array drawings and the distinctive processing, in JRDs, of perspectives aligned with the partner (at least when they were misaligned, though not counteraligned, with their partner). Finally, when describing this spatial information, speakers don't merely rely on their initial representations, but are able to use information perceptually available in the task (i.e., their degree of misalignment from their partners) to adapt descriptions appropriately.

The flexible adaptation of speakers' perspective choices, here, is consistent with the principle of least collaborative effort (Clark and Wilkes-Gibbs, 1986; Clark, 1996) in that partners shared the burden of ensuring mutual understanding and shifted their cognitive effort appropriately. When recognizing that one of them was especially likely to find the perspective-taking difficult (e.g., the Director describing the layout from a 135° offset), the other readily invested greater effort (e.g., the Matcher agreed to interpret descriptions from the Director's viewpoint).

### Integrating the partner's misaligned viewpoint with representational cues

So far, we have seen that when speakers are not instructed to adopt their partner's viewpoint and can interact freely with their partners, they may not have sufficient pragmatic motivation to organize spatial relations around a non-egocentric viewpoint. Organizing spatial relations non-egocentrically presumably requires investing cognitive effort, at least when there aren't any other spatial cues, as with the randomly configured layouts in Galati et al. (2013). In such circumstances, as we've seen, speakers can represent the partner's viewpoint relative to the spatial layout and use it later as needed. In our next study (Galati and Avraamides, in revision), we wanted to establish whether speakers *would have* sufficient pragmatic motivation to organize spatial relations around the partner's viewpoint, when that viewpoint is reinforced by additional spatial cues.

**Figure 3:**
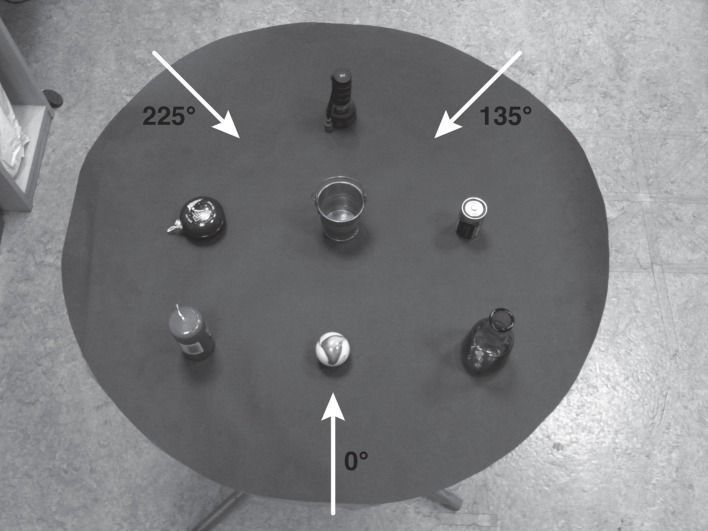
**The seven-object layout used in Galati and Avraamides (in revision), whose configuration had an intrinsic structure**. It comprised a flashlight, a yoyo, a bucket, a battery, a candle, a marble, and a vase. The arrows at 0°, 135°, and 225° represent the viewpoint that Directors and Matchers occupied at the different conditions of their relative alignment with the intrinsic structure.

The overall procedure of this study was similar: Directors first studied a spatial layout, which now had an intrinsic orientation (seven real objects were configured across a bilateral axis of symmetry, as shown in Figure 3), while either knowing their misaligned Matcher's viewpoint or not. Then, as with Galati et al. (2013), the Directors' memory of the layout was assessed through JRDs and array drawings, and finally they described the layout to their Matcher. In this experimental design, across the 24 pairs, the Director's and the Matcher's relation to the intrinsic structure of the layout differed, such that the structure was aligned with the Director, the Matcher, or neither partner. A third of the Directors studied arrays while aligned with the intrinsic structure (from 0°), and later described it to a Matcher who was offset by 135° (measured counterclockwise from 0°). Another third of the Directors studied arrays from 225° and later described it to a Matcher who was aligned with the structure (at 0°). And a final third of the Directors studied arrays again from 225° and later described to a Matcher who was offset by 135°, such that neither partner was aligned with the structure. For each group, half the Directors had known at study their Matcher's subsequent viewpoint and half of them did not. By dissociating the study from the description of the spatial layout and varying systematically the convergence of cues (i.e., whose viewpoint was aligned with the structure), we aimed to clarify how people integrate these cues as they become available.

The memory tests revealed that the preferred direction around which Directors organized spatial relations in memory depended on the convergence of cues—i.e., on whose viewpoint was reinforced by the layout's intrinsic orientation. This was most obvious by how Directors oriented their array drawings. When Directors had studied layouts while aligned with their intrinsic structure, they always drew them from their own viewpoint; it did not matter whether they knew their Matcher's subsequent viewpoint or not. When they had studied layouts while misaligned with the intrinsic structure, knowing the Matcher's subsequent viewpoint did influence how they oriented their drawings. Specifically, they were more likely to use the structure's axes (vs. their own viewpoint) as the organizing direction when knowing in advance that the Matcher would be aligned with the structure. And conversely, they were more likely to use their own viewpoint (vs. the structure's axes) when not knowing their Matcher's subsequent viewpoint. When knowing in advance that the Matcher would also be misaligned with the structure, Directors were equally likely to draw arrays from their own viewpoint or from an axis of the structure, perhaps because the intrinsic structure became more salient (relative to not knowing the Matcher's viewpoint) upon considering objects from a second oblique viewpoint.

Performance in the JRD task corroborated that Directors had indeed organized spatial relations in memory according to the orientation of their drawings. Directors who had drawn layouts aligned with the structure were faster to orient to and respond from headings aligned with the structure's axes (0°, 90°, 180°, 270°), whereas Directors who had drawn layouts misaligned with the structure (specifically from their study viewpoint of 225°), where faster to orient to and respond to from headings aligned with that viewpoint and its canonical axes (i.e., 315°, 45°, 135°).

Directors also selected perspectives strategically in their descriptions. In this study, we examined the distribution of three types of spatial expressions of theoretical interest: Director-centered, Matcher-centered, and Structure-centered ones. The latter category involved expressions that were from headings aligned with the intrinsic structure and were not person-centered (e.g., “*On the perpendicular.* You're supposed to be on one side *on the left*, and I'm on the one side of the table *on the right*.”). Overall, as shown in Table 1, Directors used reliably more Matcher-centered expressions than other types of expressions when the Matcher was aligned with the structure, and used numerically but not reliably more Director-centered expressions than Matcher-centered ones when they were the ones aligned with the structure. As with Galati et al. (2013), speakers didn't merely rely on the organization of their memories to choose the perspective of their descriptions, but rather took into account information that was perceptually available during the description phase. Although Directors used overall more Matcher-centered expressions when knowing their Matcher's viewpoint in advance, the preferred direction of Directors' memory (as reflected by their drawings) did not reliably influence their distribution of egocentric or partner-centered expressions. For example, even though most Directors who had studied layouts from 225° while not knowing their Matcher's viewpoint organized spatial information egocentrically in memory, they used overwhelmingly Matcher-centered expressions when they interacted with a Matcher who was aligned with the structure (at 0°) (see Table 1). In other words, when the convergence of available cues at the interaction strongly biases a particular perspective (e.g., when the partner's viewpoint and the structure's intrinsic alignment coincide), speakers override their initial memory representation to select the perspective of their descriptions.

Nevertheless, the advance availability of a social cue, such as the partner's viewpoint, and its relation to other cues (e.g., the intrinsic structure) can influence perspective-taking, when it highlights alternative and potentially useful perspectives for encoding and describing a spatial layout. As we have mentioned, Directors who studied layouts from 225° were relatively more likely to use the structure's axis as an organizing direction when knowing that the Matcher would be at 135° compared to not knowing the Matcher's viewpoint. Knowing in advance that neither partner was aligned with the structure influenced descriptions as well: when Directors at 225° had known in advance that Matchers would be at 135°, they used more Matcher-centered than egocentric expressions, and used numerically more Structure-centered expressions compared to not knowing the Matcher's viewpoint. Thus, knowing the partner's viewpoint while studying a layout from an oblique viewpoint can make its intrinsic organization more apparent and can influence both how speakers organize spatial information in memory and how they describe it.

Together, the findings of these two studies set the stage for a framework for how people use multiple cues, including social and representational ones, in spatial perspective-taking. Upon considering all available cues jointly and weighing them according to their salience and relevance to the task, people select the perspective reinforced probabilistically by most cues, and organize spatial information in memory or describe it to a partner accordingly. One assumption here is that people consider the perspective reinforced by multiple cues to be optimally effective in minimizing the pair's collective effort. In the next section, we examine whether in fact the perspectives pairs select make their coordination more effective.

## Coordination in spatial perspective-taking

We have so far suggested that collaborating partners select the perspective reinforced by all available cues in an effort to minimize their collective effort and maximize their efficiency of coordination—their efficiency at behaving contingently to achieve shared goal. To determine whether, in fact, people are adept at gauging which perspective would be most effective in the task, we examined two aspects of collaborative performance in our previously described studies.

The first, which tapped into pairs' efficiency on the task, was the number of conversational turns—uninterrupted stretches of speech by a Director or a Matcher—that pairs took to reconstruct a spatial layout. We took conversational turns to reflect the pairs' degree of *grounding*, or exchanging evidence about what they do or do not understand (e.g., Clark and Brennan, 1991; Clark, 1996; Brennan, 2005). Examining the pairs' turn-taking patterns enables us to identify the circumstances and description strategies that contribute to facilitated grounding: when perspective-taking strategies facilitate grounding, pairs should interact over fewer conversational turns.

The second collaborative outcome tapped into pairs' accuracy on the task: it assessed the accuracy with which Matchers, having followed the Directors' descriptions, reconstructed the spatial layouts with real objects on top of their table. Using bidimensional regression analyses we compared the Matcher's reconstruction (photographed from a bird's eye view at the end of the session) to the veridical coordinates of the original configuration. Again, when the pairs' perspective-taking strategies are effective, the Matchers' reconstructions should be less distorted.

It is important to note that what conversational partners consider to be an effective strategy is task-dependent, rather than strictly defined in terms of efficiency and accuracy. In our studies, the pairs' goal was to reconstruct layouts as accurately as possible despite lacking visual access to each other's work stations. These task-specific goals and constraints must have influenced the criterion that pairs adopted to reach the mutual belief that they had understood each other well enough for their current purposes. According to Clark and Brennan (1991), this “grounding criterion” depends both on the goals of communication (here, emphasizing accuracy) and the affordances of the communicative situation (here, lacking visibility). Thus, although an effective strategy is ideally one that maximizes efficiency in terms of turn-taking while also yielding high accuracy on the resulting reconstruction, in some circumstances, efficiency and accuracy may be dissociated if weighted differently by the task's goals.

### Social and representational cues shape grounding

For the pairs who reconstructed randomly configured layouts (Galati et al., 2013), knowing the partner's viewpoint at learning helped their subsequent efficiency in some circumstances. Specifically, pairs took numerically fewer turns to complete the reconstruction of the layout when they had mutually known in advance that they would be misaligned by the oblique 135° than by the other, orthogonal offsets (see Galati and Avraamides, 2012). This counterintuitive pattern makes sense insofar as the Directors' description strategies were suitable. As we've reported in the previous section, in Galati et al. (2013), at the oblique and more computationally demanding offset of 135°, Directors showed a strong preference for describing layouts from their own perspective, frequently upon their Matcher's prompting. This strategy turned out to be beneficial in alleviating their collective effort, as reflected by their conversational turns. In fact, as Directors used greater proportions of egocentric expressions when knowing their Matcher's viewpoint in advance, pairs took reliably fewer turns to reconstruct the layout.

In this article, we present some analyses not reported elsewhere, on the efficiency of pairs who reconstructed layouts that had an intrinsic structure (from the corpus of Galati and Avraamides, in revision). In these circumstances, the number of turns that pairs took to complete the task was determined primarily by the alignment of the two partners' viewpoints relative to the intrinsic structure at the description, *F*_(2, 18)_ = 4.44, *p* < 0.05. Here, advance knowledge of the partner's viewpoint did not influence significantly the number of turns pairs took to reconstruct the layout (*p* = 0.99) and did not interact with the partners' alignment with the structure (*p* = 0.98). Partners were the least efficient when neither of them was aligned with the intrinsic structure during the description: in that scenario they took an average of 259.25 turns (*SD* = 125.29), whereas they took an average of 114.88 turns (*SD* = 66.87) when the Matcher was aligned with the intrinsic structure and 166.75 turns (*SD* = 68.27) when the Director was aligned with it. Indeed, compared to when neither partner was aligned with the structure, pairs took significantly fewer turns when the Matcher was aligned with it, 95% CI (−247.49, −41.26), *p* < 0.01, and marginally more so when the Director was, 95% CI (−195.62, 10.62), *p* = 0.08.

Together, the patterns of turn-taking in the two experiments underscore the flexibility with which people use all available cues to select a spatial perspective in a joint task. When the spatial layout does not afford any representational cues (as when it is randomly configured), the a priori availability of a social cue, such as the partner's subsequent viewpoint, can enable partners to recognize when coordinating a perspective would be difficult for the partner bearing greater responsibility for mutual understanding and to agree on a perspective that alleviates that partner's cognitive demands. With turns as a proxy of partners' collaborative effort, these mutually agreed-upon strategies can make their interactions more efficient. On the other hand, when the spatial layout affords an intrinsic organization, its alignment relative to each partner during the interaction is what influences most the efficiency of coordination. In general, interactions are more efficient when the orientation of structure of the layout converges with one of the partner's viewpoints than when it does not. Even though pairs were misaligned by a smaller offset when neither of them was aligned with the structure (by 90°) compared to when one of them was aligned with the structure (in which case their offset was 135°), the process of coordination was lengthier: thus, it was their relation to the intrinsic structure, not their misalignment from each other, that influenced their efficiency. We will return to this point in the final section of our article.

Thus far, we have seen that pairs generally adopt strategies that make their coordination more efficient in terms of the number of conversational turns they take to complete their joint task. When the layout provides intrinsic cues that coincide with a given partner's perspective, speakers describe the layout from that person-centered perspective and this strategy is effective. When the layout does not provide such intrinsic cues, a priori information about the partners' relative viewpoints helps determine which perspective is optimal for the speaker—adopting that perspective is an effective strategy.

### Social and representational cues shape the pairs' accuracy on the task

Although the availability and convergence of various cues facilitated performance in terms of the efficiency of dialogs as reflected by turn-taking, it didn't facilitate performance in the same way in terms of accuracy on the task. We assessed accuracy by examining the bidimensional regression coefficient (*BDr*), which estimates the goodness-of-fit between the tabletop reconstructions and the actual coordinates of the arrays, thus capturing unsystematic error in reconstructions when systematic biases are accounted for. We also examined the rotation parameter (θ), which indicates the degree to which the tabletop reconstruction was rotated relative to the studied array, thus capturing a potential systematic bias in the reconstructions.

In our study with randomly configured layouts, the only reliable finding from examining the Matcher's tabletop reconstructions was that the relationships among objects became more distorted as Directors used more Matcher-centered expressions (see Galati and Avraamides, 2013). This could be due to Directors inadvertently introducing more inaccuracies in descriptions when computing spatial relations and selecting spatial terms from a non-egocentric perspective. This possibility is supported by the fact that, when partners were offset by 180° and Directors could more easily map egocentric spatial terms to partner-centered ones (e.g., *my left = your right*), the reconstructed layouts were less distorted than at the offsets of 90° and 135°.

In our study with layouts with an intrinsic structure, our new analyses reported here reveal a somewhat different pattern. Although the *BDr* was not reliably correlated with any of the three main types of expressions (Director-centered, Matcher-centered, Structure-centered), pairs reconstructed were less distorted layouts as Directors used greater proportions of Matcher-centered expressions with Matcher's aligned with the layout's intrinsic structure (Pearson's *r* = 0.83, *p* < 0.05). As we have shown in Galati and Avraamides (in revision), in this alignment condition Directors adopted the strategy of describing layouts from their Matcher's viewpoint, using overwhelmingly Matcher-centered expressions. This strategy was therefore effective, not only in terms of reducing the number of turns (see previous subsection), but also in terms of yielding less distorted reconstructions, underscoring that there was no speed-accuracy tradeoff in pair's efficiency. In general, reconstructions did not become more distorted as pairs interacted over fewer turns (Pearson's *r* = −14, *p* = 0.52), suggesting that pairs upheld the goal of the task to reconstruct layouts that were as accurate as possible.

Nevertheless, pairs demonstrated a systematic bias in rotating the spatial layout when its intrinsic structure was aligned with the Matcher during the description. For reconstructions in that condition, the average rotation parameter was θ = 1.94, *r* = 0.24. Individual θ 's were not uniformly distributed around 0° (*V* = 1.88, *p* = 0.17). On the other hand, for reconstructions in the aligned-with-Director condition, the average rotation parameter was θ = 9.93, *r* = 0.97, 95% CI [−3.22, 23.08], and individual scores were uniformly distributed around 0°, *V* = 6.66, *p* < 0.001). This was also the case for reconstructions in the aligned-with-Neither condition, θ = −8.07, *r* = 0.94, 95% CI [−24.23, 8.09], *V* = 7.47, *p* < 0.001.

To summarize, although collaborating partners are successful at selecting perspectives that increase their efficiency, by minimizing their collective effort in terms of the length of their interaction, these perspectives don't always make them accurate on the task. In particular, decrements in accuracy seem to arise when speakers describe spatial information from the partner's viewpoint, especially when the configuration does not afford an intrinsic structure. When the configuration does afford an intrinsic structure, adopting the partner's perspective when it is reasonable to do so (when the partner is aligned with the structure) may be effective in some ways (e.g., reducing the length of the interaction, reducing distortion in the reconstructions) but not others (e.g., eliminating systematic rotational biases).

## A framework for flexible perspective-taking in spatial tasks

Our findings contribute to a framework for flexible perspective-taking that captures several of the nuanced ways in which speakers reason and coordinate in spatial tasks. In our framework, perspective-taking is flexible insofar as speakers consider all available cues—both social and representational—and weigh them according to their salience and relevance to the task to select the most effective perspective. This probabilistic weighing of cues distinguishes our framework from others that ascribe precedence to egocentric experience (Shelton and McNamara, 2001) or intrinsic structure (Mou and McNamara, 2002). Another consequence of this simultaneous weighing of multiple cues is that a single cue, such as the partner's viewpoint, may require further reinforcement from other cues to be adopted as an organizing direction in spatial memory. Pragmatic motivation from explicit instructions (Shelton and McNamara, 2004) or from the intrinsic structure (Galati and Avraamides, in revision) can supply such reinforcement. It also suggests that the misalignment between partners does not on its own reflect the computational demands of perspective-taking; instead, as we will argue, misalignment can lead to appropriate attributions about each partner's cognitive demands only in conjunction with other cues. Our framework's proposal that people use multiple, weighted cues extends to non-social spatial perspective-taking as well, affording predictions for which perspective or organizing direction they will select, even when reasoning for themselves.

That interacting partners take into account take each other's relative cognitive demands when selecting a perspective further underscores the flexibility of perspective-taking. Through this process, they determine the most effective perspective to use both for organizing information in memory and for describing it to one another. As we will discuss, in determining their relative cognitive demands, people take into account the collective effort invested across all phases of their joint task, from learning to the interaction.

In our framework, perspective-taking is also flexible in the sense that speakers don't rely blindly on their memories when selecting the perspective of their descriptions. Instead, they use perceptual information from the communicative information (e.g., about their partner's viewpoint), even if this hadn't been available in advance. In other words, they use both a priori and incrementally unfolding cues to update their attributions about which perspective would be optimal. Their assessment for what constitutes an effective perspective that would minimize their collective effort and maximize their performance depends on the grounding criterion they adopt in light of task's goals and constraints.

Finally, perspective-taking is flexible insofar as reflects the general flexibility of the cognitive system. Our framework considers partner-specific adaptation to emerge from ordinary cognitive processes acting on ordinary memory representations, whether spatial or episodic ones. As such, the principles of our framework—that speakers consider a confluence of cues, whether available perceptually or a priori, aiming to minimize collective effort—hold not just for spatial perspective-taking, but for conversational perspective-taking more broadly.

Below we expound further on the main characteristics of this framework and the insights that follow from it.

### People consider simultaneously social and representational cues

During the course of perspective-taking, people consider various sources of information, including social cues (e.g., the availability of the partner's viewpoint), representational spatial cues (e.g., the layout's intrinsic structure), and egocentric biases (e.g., based one's own learning viewpoint). When multiple cues are available, people consider their confluence, weighing them according to their salience and relevance to the task.

In weighing multiple cues, people in collaborative tasks have to appraise the relative cognitive demands on each partner in order to select the perspective that minimizes their collective effort. An assumption here is that the perspective that is reinforced by the greatest number of cues or by the most salient cues is the most effective and thus preferable for encoding spatial information in memory and in language. Indeed, as we have shown, converging social and representational cues (e.g., the alignment of the layout with a given partner's viewpoint) motivate the use of a given perspective as the preferred orientation in memory and in descriptions.

Critically, a social cue, such as the availability of the partner's viewpoint, may not be sufficient on its own to shape the organizing direction of spatial memories (Galati et al., 2013) since organizing spatial relations around that viewpoint is costly and is unnecessary when pairs can interact freely and can correct misunderstandings (cf., Shelton and McNamara, 2004). As we have shown, in free dialogs, the partner's viewpoint may simply be encoded in memory. However, when this social cue converges with other cues (e.g., the layout's intrinsic structure), it can be used as the preferred direction of spatial representations at no discernible cost, despite being non-egocentric.

The intrinsic orientation of a spatial configuration is therefore one factor that contributes to adopting a non-egocentric viewpoint around which to organize spatial relations in memory. Related findings have led other researchers to propose that the intrinsic orientation of a layout is the dominant factor determining the preferred direction around which to organize information in memory (Mou and McNamara, 2002). However, in our framework, rather than ascribing precedence to particular cues, all available cues are weighted probabilistically according to task-specific demands. (Indeed, in Galati and Avraamides, in revision, Directors didn't invariably organize information in memory according to the configuration's intrinsic structure.) When multiple cues that are relevant to the task reinforce a particular viewpoint, that viewpoint is more likely to be adopted. Thus, when the orientation of the structure converges with one's own viewpoint, people opt for that egocentric viewpoint, whereas when it converges with their partner's viewpoint, they opt for their partner's viewpoint.

A final observation is that social cues can be combined not only with other types of non-social information (e.g., representational cues), but also with other types of social cues. A contextual cue concerning the partner (e.g., his misalignment from the speaker) can interact with an attributional cue about the partner (e.g., concerning his spatial abilities). For example, when a speaker describes a spatial layout to a partner misaligned by a relatively difficult offset (e.g., the oblique 135°), she may use more partner-centered expressions if she perceives him to have relatively poor spatial abilities, but more egocentric ones if she perceives him to have relatively good spatial abilities. Such predictions following from our framework can be explored in future research.

### People consider the cognitive demands of perspective-taking for both partners

Our framework accommodates and is compatible with *the principle of least collaborative effort* (Clark and Wilkes-Gibbs, 1986; Clark, 1996)—the view that, in sharing responsibility for mutual understanding, conversational partners adapt their behavior in ways that aim to minimize their collective effort and facilitate their coordination.

In collaborative spatial tasks, the relative cognitive demands of perspective-taking on each partner motivate the perspective from which people encode or describe spatial information. Critically, the partner's viewpoint can influence the process of estimating their respective perspective-taking demands, as soon as it becomes available—whether at encoding or at the interaction.

In several real-world scenarios, people first have to commit certain spatial information to memory and convey it to someone else later, as for example, on a road trip when the co-pilot studies the route to the destination on a map and then gives directions from memory to the driver. In such situations, our framework posits that, to gauge their and their partner's relative cognitive demands, speakers must consider the cognitive effort they would invest in total, both when encoding the information and when describing it. Speakers must therefore estimate whether investing additional cognitive effort at encoding would yield savings in the effort they would expend later, when coordinating with their partner.

Having information about the upcoming interaction available in advance enables speakers to better anticipate the perspective most effective during the interaction and to adapt their encoding strategies accordingly. In our work, when speakers knew in advance that their partner's viewpoint was aligned with the layout's intrinsic orientation, they were more likely to adopt it as an organizing direction at encoding. Organizing spatial relations according to the partner's viewpoint made sense in terms of minimizing subsequent effort: speakers judged that this would be an effective perspective from which to describe the layout since the partner would not have to unpack the mappings of spatial expressions. Indeed, when the partner was aligned with the structure, speakers used overwhelmingly partner-centered expressions and pairs were the most efficient, at least in terms of their conversational turns.

Nonetheless, the availability of the partner's viewpoint alone, without the reinforcement of intrinsic spatial cues is not sufficient motivation, in free dialogs, to invest in organizing spatial relations around their partner's viewpoint. As we have seen, when speakers studied randomly configured layouts, they simply represented that viewpoint in memory in order to use it later, as needed. Despite not having invested the effort to encode such layouts from their partner's viewpoint, speakers could still adapt their descriptions upon considering the relative cost of perspective-taking based on their misalignment (see the subsection on the right column for a more detailed discussion of the factors contributing to the cost of perspective-taking). For instance, speakers could still adopt their partner's viewpoint in descriptions when perspective-taking was relatively easy for them (e.g., at small or canonical offsets). And when perspective-taking was relatively difficult (e.g., at oblique offsets), speakers would opt for their own perspective in descriptions. Their partner's endorsement of this strategy indicates that pairs mutually agree to reduce the cognitive demands of the speaker, who in this context was encumbered by the greatest effort due to having to retrieve and describe spatial relations from memory.

People's dynamic and sophisticated adaptation of perspective choices suggests that they seek perspectives that are optimally effective in minimizing their effort, not just when collaborating, but also when investing cognitive resources in preparation for that collaboration. This is a novel elaboration of the principle of least collaborative effort.

### People use flexibly a priori and perceptually available information

The above discussion, regarding the cognitive demands at encoding and at the interaction, underscores the dissociation between the perspective of spatial descriptions and of the spatial memories supporting those descriptions. Our work demonstrates that speakers don't merely rely on the organization of their memories to select how to describe spatial relations, but instead also use information that is perceptually available in the interaction. A contextual social cue, such as the partner's viewpoint, can shape descriptions even it had been unavailable at encoding and thus not incorporated in speakers' memory representations.

For example, in Galati et al. (2013), when the partner's viewpoint wasn't available at study speakers didn't necessarily use more egocentric expressions at the description, and conversely, when the partner's viewpoint was available at study speakers didn't necessarily use more partner-centered descriptions. Instead, speakers' description strategies were guided by contextual cues they encountered at the interaction: seeing that the partner was misaligned by a relatively small offset led to more frequent use partner-centered descriptions, whereas seeing that the partner was misaligned by an oblique offset led to more frequent use of egocentric expressions.

Similarly, in Galati and Avraamides (in revision), the contextual social cue of the partner's viewpoint shaped descriptions even when its relation to the layout's structure was unknown at encoding. Overall, the organization of speakers' memories (as reflected by the orientation of their array drawings) didn't reliably influence their descriptions. For instance, despite most frequently encoding a spatial layout egocentrically when having studied it from a viewpoint oblique to its structure (225°) without knowing the partner's viewpoint, speakers overwhelmingly used partner-centered expressions upon encountering a partner aligned with the structure at the description.

Together, these findings suggest that speakers carefully attend to contextual social cues—partner-specific information that is perceptually available in the social situation—and use this information readily. As a result, they may override their perspective preferences for encoding the spatial information. This view is compatible with findings that people don't always adhere to the organizing direction of their memories when it conflicts with perceptual evidence, but use instead both sources of information to select the perspective of their descriptions (Li et al., 2011). Thus, the organization of spatial memories does not dictate how spatial information is subsequently described. Descriptions are also guided by perceptual information, which partners use to determine the optimal perspective for the collaborative task.

### People don't assess the relative difficulty of perspective-taking only based on their misalignment

There have been some incongruent findings concerning the offsets at which spatial perspective-taking is most difficult in collaborative tasks. In a study that involved interpreting another's spatial descriptions, listeners incurred a greater processing cost as the degree of misalignment from their partner increased (Duran et al., 2011). On the other hand, some studies focusing on production reported similarities in speakers' descriptions across misaligned offsets: with misaligned partners, speakers used partner-centered expressions with comparable frequency, regardless of the degree of misalignment (Schober, 1993, 1995; Mainwaring et al., 2003). This was taken as evidence against a mental rotation model of perspective-taking, and in favor of a categorical distinction between reasoning from an egocentric vs. a non-egocentric perspective (Schober, 1995). However, in all of these studies, real or assumed partners were misaligned by orthogonal offsets (90°, 180°, 270°). This methodological feature may limit our understanding of when perspective-taking is most demanding since, according to McNamara (2003), perspectives aligned with canonical axes can be facilitated relative to oblique ones.

Our findings are line with McNamara (2003) view, since when no intrinsic cues were available speakers opted for egocentricism when they were misaligned by 135° from their partners: they were more likely to use egocentric expressions at 135° than at 90°, but no more likely (and, in fact, marginally less likely) to do so at the maximum offset 180°. These findings suggests that this oblique viewpoint is more computationally demanding, at least when producing spatial descriptions (though we find converging evidence from the interpretation of spatial descriptions in ongoing work in our lab).

Our findings offer a further caveat: it is not misalignment alone that ultimately determines the difficulty of perspective-taking, but its combination with other cues. In our study with layouts with an intrinsic structure, speakers made different description choices depending on the alignment of the structure with either partner, despite the partners' misalignment remaining the same. Directors who were at 0° with Matchers at 135° overall opted for their own perspective in descriptions, presumably because reasoning from a perspective oblique to the structure (and their own) was computationally more difficult. However, Directors who were at 225° with Matchers at 0° (also a 135° offset) readily opted for their partner's perspective.

In sum, people do not simply mentally rotate a spatial configuration in order to consider their partner's viewpoint. It is not the case that as the misalignment between partners increases perspective-taking becomes more difficult. Adopting the partner's viewpoint when the partner is misaligned by an oblique offset is generally more difficult than canonical offsets, though not when it is reinforced by other representational cues. The misalignment between partners determines the relative difficulty of perspective-taking for each partner in conjunction with other cues.

### People select perspectives that lead to more efficient but not always more accurate performance

As we have noted, the adaptation we documented in our studies is consistent with a principle governing human interaction, whereby conversational partners seek to minimize their collective effort and maximize the efficiency of their coordination (Clark and Wilkes-Gibbs, 1986; Clark, 1996). Overall, attributions about the partner's ability to contribute to mutual understanding, enabled by either a priori or perceptual information, lead to strategies that improve task performance. In our studies, recognizing which perspective would be optimal for a particular set of circumstances led to description strategies that were successful at reducing collective effort. Despite the high grounding criterion that pairs had to adopt, given that instructions emphasized accuracy and that speakers could not visually monitor their partner's progress in reconstructing the layout, speakers still managed to select strategies that made interactions efficient.

For instance, pairs took fewer turns to reconstruct randomly configured layouts when they knew in advance that they would be misaligned by an oblique and presumably computationally demanding offset, compared to other orthogonal offsets (Galati and Avraamides, 2012). Under those circumstances, pairs recognized that adopting the perspective of the speaker would be beneficial and were more likely to explicitly agree on that perspective in advance. Thus, when the spatial layout does not afford intrinsic cues, a priori information about the partners' cognitive demands (derived from their relative viewpoints) helps pairs select strategies that make the interaction efficient.

When the layout does afford intrinsic cues, considering the relation of those spatial cues to social cues was critical to determining the optimal perspective. As we've found, interactions took longer in terms of turn-taking when intrinsic cues were not aligned with either partner compared to when they were. And when intrinsic cues converged with the perspective of the partner (vs. the speaker), interactions were somewhat more efficient. This is likely because it was easier for partners to interpret partner-centered expressions (which speakers used almost exclusively when the structure was aligned with the partner) than speaker-centered expressions (which speakers used at greater proportions when they were the ones aligned with the structure).

Nevertheless, although partners made reasonable assumptions about which perspective would be optimal to adopt and although these perspectives minimized their collective effort in terms of their conversational turns, they didn't necessarily improve all aspects of performance on the task. In terms of accuracy, we've found that when the partner was aligned with the layout's structure, reconstructions exhibited a significant rotational bias relative to the other alignment conditions, despite being significantly less distorted the more partner-centered expressions were used. Thus, adopting the partner's perspective in this scenario was an effective strategy in most but not all outcomes.

Adopting the partner's perspective when layouts did not afford an intrinsic structure was actually detrimental to accuracy: reconstructions were more distorted as speakers used more partner-centered expressions. This distortion was curbed somewhat when partners were counteraligned, perhaps because the straightforward mappings of egocentric to other-centered expressions (e.g., *my* left = your right) made it easier for speakers to provide more accurate descriptions, or for partners to interpret speakers' descriptions in the intended way.

Altogether, even though in our studies accuracy was prioritized in pairs' joint goal, it wasn't always achieved perfectly. Whether the source of inaccuracies resides in the speakers' descriptions or the addressees' interpretations remains unresolved. Future research could clarify this by examining task performance against the qualitative content and structure of the pairs' dialogs, beyond just the proportions of speakers' spatial expressions (e.g., high-level description strategies, such separating the table in quadrants). Another methodological consideration for future studies would be to include measures of spatial ability for both collaborating partners. Accounting for some of the variability arising from individual differences in spatial ability can help distinguish whether decrements in accuracy are due to speakers' poor recall and inadequate descriptions or due to partners' misinterpretation of otherwise accurate descriptions. Such efforts would inform the dynamic coupling of partners behaving contingently in joint spatial tasks.

### Perspective-taking beyond spatial tasks

Our framework for spatial perspective-taking reflects the general flexibility of the cognitive system; it is not intended as a framework specialized for or limited to spatial perspective-taking. Our view is that coordination in spatial perspective-taking is governed by some of the same principles as non-spatial perspective-taking—when people consider their conversational partner's conceptual construal, their knowledge, or agenda (see Schober, 1998).

To determine the similarity of their conceptual perspectives, people routinely have to consider what they have in common ground with their conversational partner and to tailor how to produce or interpret utterances. Discrepancies in perspective are especially apparent when there are asymmetries in the partners' respective knowledge or ability, as when one interacts with a non-native speaker (Bortfeld and Brennan, 1997) or a novice (Isaacs and Clark, 1987). Indeed, when people share the same perspective (whether conceptual or physical), it can be trivially easy to adopt the partner's perspective; people can perform generic linguistic or behavioral adjustments (benefiting themselves), rather than adjustments that are specifically designed for their partner (Brown and Dell, 1987; Dell and Brown, 1991). Investigations of partner-specific adaptation should therefore dissociate the perspectives of speakers and their partners (see Keysar, 1997).

Our empirical undertaking to unveil the relation between linguistic perspective choices and the underlying spatial memories that support them is compatible with a memory-based view of partner-specific adaptation. This view considers linguistic and behavioral adjustments to the partner to emerge from cognitive constraints acting on memory-dependent processes (Metzing and Brennan, 2003; Pickering and Garrod, 2004; Horton and Gerrig, 2005). Specifically, shared experiences with a partner and partner-specific associations are considered to be represented in memory and accessed through ordinary processes, such as resonance with combinations of cues in working memory, influencing behavior accordingly (Horton and Gerrig, 2005). In this view, failures in perspective-taking occur when relevant information about the partner isn't available early enough (Kraljic and Brennan, 2005), when complex inferences about the partner have not yet been made (Gerrig et al., 2000), when executive functioning is taxed (Brown-Schmidt, 2009), or under time pressure (Epley et al., 2004).

Our own findings underscore that simple but relevant cues about the partner (e.g., the partner's location in space, their relation to a configuration's intrinsic structure) can indeed be represented and used to compute the relative difficulty of reasoning from their perspective, consequently determining linguistic choices. This is also in agreement with proposals that when information about the partner is readily available, can be represented simply or computed unambiguously, it can influence language processing at no discernible cost, relative to egocentric processing (Brennan and Hanna, 2009; Galati and Brennan, 2010, 2013).

Our framework is also in line with constraint-based models of language processing (e.g., MacDonald, 1994; Tanenhaus and Trueswell, 1995; McRae et al., 1998). According to constraint-based models, information from various sources, including the discourse context, within-sentence structural, lexical biases, and even information about the partner (e.g., Hanna et al., 2003; Brown-Schmidt and Hanna, 2011), is integrated probabilistically and in parallel to shape the interpretation of utterances, and presumably also speech plans. Similarly, in computational models of perspective-taking, attributions about the partner can be represented as control parameters that can alter behavior (e.g., Duran and Dale, 2013). Other computational accounts also underscore that language processing is adaptive by demonstrating that language users update probability distributions of relevant discourse features (e.g., syntactic structures) as new linguistic evidence becomes available (Fine et al., 2010).

In our work, we've demonstrated that in spatial tasks people indeed use all relevant information from various sources, whether it becomes available at encoding or at collaboration, to form attributions about each partner's relative cognitive effort, which they can update during the course of the interaction and tailor their behavior. This relevant information can include contextual social cues, such as the partner's location in space, or attributional cues, such as beliefs or expectations about the partner's spatial abilities. Such social cues may combine with other cues—intrinsic or functional properties of the objects, the intrinsic structure of the layout or the surrounding environment, one's egocentric viewpoint, and explicit instructions—to determine perspective choices in a constraint-based fashion.

Such an approach departs from proposals that have, on the one hand, acknowledged that the organization of spatial memories depends on the contribution of several cues, but on the other hand, held that certain cues are dominant (Shelton and McNamara, 2001; Mou and McNamara, 2002). Neither egocentric experience (Shelton and McNamara, 2001) nor the intrinsic structure of a spatial configuration (Mou and McNamara, 2002) necessarily need to carry the greatest weight across all tasks. Instead they interact with other weighted parameters, including attributional and contextual cues about the partner.

Finally, our framework for the flexible processing of multiple cues can be extended to non-interactive spatial perspective-taking tasks. We propose that even in non-social situations where people have to imagine adopting different perspectives in space (as when imagining how our redecorated living room would look from different vantage points), the preference for or ease of adopting particular perspectives depends on the confluence of weighted relevant cues.

### Conclusion

In this article, we have emphasized the centrality of social cues in spatial perspective-taking and have outlined a framework for flexible adaptation of memory and behavior in collaborative spatial tasks. Studying spatial perspective-taking by focusing entirely on individual processes overlooks the ubiquitous and remarkable ability with which people coordinate with one another in a range of everyday activities. The findings emerging from our experimental work underscore people's ability to appraise both social and other representational cues to select the perspective that would be optimal for minimizing their collective effort. Thus, information about the partner (whether derived from the visual context, or from inferences or prior expectations), alongside other cues, can shape how spatial relations are organized in memory and whose perspective is adopted in descriptions. We have argued that this adaptation involves weighing cues according to their relevance and salience to the task, similar to constraint-based approaches, and selecting the perspective most reinforced by the summated contribution of those cues. Moreover, cues are factored into this process whenever they become available—whether through perceptual evidence or advance knowledge. This highlights the flexibility with which people convey information accessed from spatial memory: rather than merely relying on their memory's organization, their assessment of task-specific demands is updated by incoming cues.

Partner-specific adaptation in spatial tasks emerges from processes comparable to those governing non-spatial perspective-taking. This holds both for the principles that regulate the social dynamics of interacting partners (e.g., the principle of least collaborative effort), and for the general cognitive architecture that supports adopting spatial and conceptual perspectives other than one's own. When executive functioning is overloaded, or when relevant cues aren't readily available or easily computed, the ability to appraise the optimal perspective for the joint task is compromised. Partners in perspective-taking tasks—spatial and non-spatial—consider multiple sources of information to make attributions about their respective ability to contribute to mutual understanding. According to these attributions, they adapt how they represent partner-specific information in memory and how they coordinate in dialog.

### Conflict of interest statement

The authors declare that the research was conducted in the absence of any commercial or financial relationships that could be construed as a potential conflict of interest.
